# Laparoscopic lavage for Hinchey III perforated diverticulitis: factors for treatment failure in two randomized clinical trials

**DOI:** 10.1093/bjs/znad114

**Published:** 2023-05-18

**Authors:** Najia Azhar, Daniël Lambrichts, Johan Lange, Sheraz Yaqub, Tom Øresland, Johannes Schultz, Willem Bemelman, Pamela Buchwald

**Affiliations:** Department of Surgery, Skåne University Hospital, Malmö, Sweden; Department of Clinical Sciences Malmö, Lund University, Malmö, Sweden; Department of Surgery, Erasmus University Medical Centre, Rotterdam, The Netherlands; Department of Surgery, University Medical Centre Amsterdam, AMC, Amsterdam, The Netherlands; Department of Surgery, Erasmus University Medical Centre, Rotterdam, The Netherlands; Faculty of Medicine, Institute of Clinical Medicine, University of Oslo, Oslo, Norway; Department of Gastrointestinal Surgery, Oslo University Hospital, Oslo, Norway; Faculty of Medicine, Institute of Clinical Medicine, University of Oslo, Oslo, Norway; Faculty of Medicine, Institute of Clinical Medicine, University of Oslo, Oslo, Norway; Department of Digestive Surgery, Akershus University Hospital, Lørenskog, Norway; Department of Surgery, University Medical Centre Amsterdam, AMC, Amsterdam, The Netherlands; Department of Surgery, Skåne University Hospital, Malmö, Sweden; Department of Clinical Sciences Malmö, Lund University, Malmö, Sweden

## Abstract

**Background:**

The Scandinavian Diverticulitis (SCANDIV) trial and the LOLA arm of the LADIES trial randomized patients with Hinchey III perforated diverticulitis to laparoscopic peritoneal lavage or sigmoid resection. The aim of this analysis was to identify risk factors for treatment failure in patients with Hinchey III perforated diverticulitis.

**Methods:**

This was a *post hoc* analysis of the SCANDIV trial and LOLA arm. Treatment failure was defined as morbidity requiring general anaesthesia (Clavien–Dindo grade IIIb or higher) within 90 days. Age, sex, BMI, ASA fitness grade, smoking status, previous episodes of diverticulitis, previous abdominal surgery, time to surgery, and surgical competence were all tested in univariable and multivariable logistic regression analyses using an interaction variable.

**Results:**

The pooled analysis included 222 patients randomized to laparoscopic lavage and primary resection (116 and 106 patients respectively). Univariable analysis found ASA grade to be associated with advanced morbidity in both groups, and the following factors in the laparoscopic lavage group: smoking, corticosteroid use, and BMI. Significant factors for laparoscopic lavage morbidity in multivariable analysis were smoking (OR 7.05, 95 per cent c.i. 2.07 to 23.98; *P* = 0.002) and corticosteroid use (OR 6.02, 1.54 to 23.51; *P* = 0.010).

**Conclusion:**

Active smoking status and corticosteroid use were risk factors for laparoscopic lavage treatment failure (advanced morbidity) in patients with perforated diverticulitis.

## Teaser

This study analysed a prospectively collected cohort of patients with Hinchey III diverticulitis from two large randomized trials (SCANDIV and LOLA), aiming to find risk factors for treatment failure. The results showed that only active smoking status and corticosteroid use in the laparoscopic lavage group were risk factors for treatment failure.

## Introduction

Complicated diverticulitis is characterized by perforation, stenosis, fistulas, and obstruction^1^. The extension of abdominal contamination owing to perforation is classified according to Hinchey^2^. Initially, the classification was based on surgical findings, but the improvement in radiological diagnostics, especially CT, has resulted in localized abscesses (Hinchey grade I and II) often being treated with antibiotics with or without percutaneous drainage. Surgery is indicated in patients with perforation causing abdominal contamination and purulent or faecal peritonitis (Hinchey grade III or IV respectively), particularly if the patient develops sepsis^3,4^.

The role of laparoscopic lavage as means of treating Hinchey III disease has been debated and investigated. Recently, the results of three RCTS^5–7^ comparing laparoscopic lavage with primary resection for Hinchey grade III perforated diverticulitis have been published. Meta-analyses^8^ of these studies showed that laparoscopic lavage in patients with Hinchey III diverticulitis is associated with the need for repeated interventions because of ongoing sepsis, but no difference in mortality rates were found and fewer patients undergoing laparoscopic lavage had a stoma at 1-year or longer follow-up^5,9–12^. Moreover, laparoscopic lavage is more easily performed than primary resection, is cheaper, and long-term follow-up has shown no differences in terms of morbidity or mortality^11,13^. However, laparoscopic lavage fails in about one in five patients, so identifying preoperative risk factors for treatment failure would be helpful. Until now, risk factors for treatment failure have been explored only in a few heterogeneous studies^14–16^.

The SCANDIV trial^6^ and the LOLA arm of the LADIES trial^5^ randomized a total of 222 patients with Hinchey III diverticulitis to either laparoscopic lavage or primary resection (116 and 106 respectively). The aim of this study was to identify potential risk factors for treatment failure in patients with Hinchey III perforated diverticulitis in this large, prospectively collected, randomized study population.

## Methods

Both studies were approved by national ethics boards initially, and a supplement to the primary ethics approval was sent in and approved (2021-06755-02) in Sweden and Norway. In the Netherlands, additional approval was not considered necessary by the Medical Ethics Review Committee of the Academic Medical Centre.

Individualized data were retrieved from SCANDIV and the LOLA arm of the LADIES trial. All data were collected prospectively. The SCANDIV trial was designed as a pragmatic, two-armed, open-labelled, multicentre, superiority randomized trial. Patients were enrolled in 21 surgical units (9 in Sweden and 12 in Norway)^6^. Primary enrolment was conducted between 5 February 2010 and 28 June 2014. Included patients were older than 18 years, and had a clinical suspicion of perforated diverticulitis and a need for emergency surgery. The LOLA study was a parallel-group, multicentre, randomized, open-label trial conducted in 30 hospitals in Belgium (1), Italy (1), and the Netherlands (28)^5^. Primary enrolment was undertaken between 1 July 2010 and 22 February 2013. The inclusion criterion was perforated purulent diverticulitis at laparoscopy. Patients with faecal peritonitis, age over 85 years, high-dose corticosteroid use (at least 20 mg daily), and haemodynamic instability were excluded.

Patients were evaluated on an intention-to-treat basis. All factors that may predict treatment failure, defined as complications of at least Clavien–Dindo grade IIIb (requiring general anaesthesia), were analysed^17^. These included patient characteristics, and perioperative and postoperative variables such as age, sex, BMI, ASA fitness grade, smoking (yes or no), and immunosuppression (defined as use of immunosuppressive medication in SCANDIV and use of corticosteroids (less than 20 mg daily) in LOLA). Corticosteroid use referred to oral intake. Furthermore, duration of operation, time to surgery, and duration of surgical ICU stay were assessed. Only factors that were registered in both studies were analysed.

### Statistical analysis

Logistic regression in a two-step approach was used to investigate risk factors for failure and compare potential risk factors between operation types. ORs with 95 per cent confidence intervals and *P* values were calculated. In the first step, potential risk factors and interaction terms between potential risk factors and operation type were analysed in separate models for each risk factor. Risk factors with *P* < 0.100 for at least one operation type were included in a multiple logistic regression model. In both steps, all analyses were adjusted for study (SCANDIV or LOLA). All analyses were performed using the statistical software SAS^®^ version 9.4 (SAS Institute, Cary, NC, USA).

## Results

**Fig. 1:**
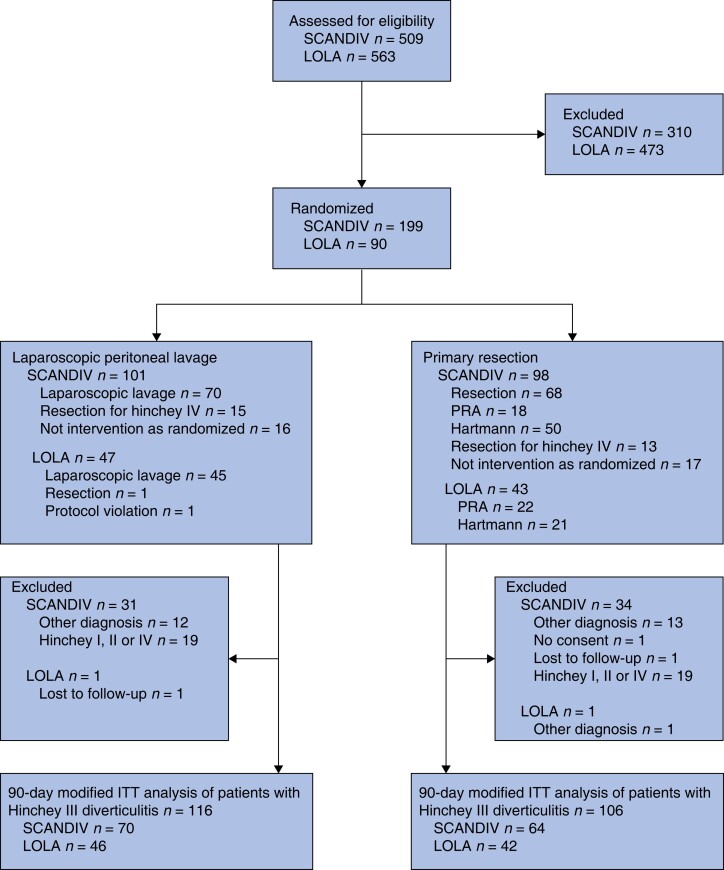
Study flow chart PRA, primary resection with anastomosis; ITT, intention to treat.

A total of 222 patients with Hinchey III perforated diverticulitis were included in the pooled analysis, of whom 116 were randomized to the laparoscopic lavage group and 106 to the primary resection group (*Fig. 1*). Baseline characteristics for all patients, as well as those for each separate study population and procedure, are shown in *Tables 1–3*. There were no significant differences between patients in the two studies, except greater cortisone use in SCANDIV and more smokers in the LOLA study.

**Table 1:** Baseline characteristics of all patients with Hinchey III diverticulitis

	All patients (*n* = 222)	SCANDIV (*n* = 134)	LOLA (*n* = 88)
Age (years), mean(s.d.)	64.4(13.7)	65.2(14.5)	63.1(12.5)
Sex ratio (M : F)	125 : 97	74 : 60	51 : 37
BMI (kg/m^2^), mean(s.d.)	26.7(5.0)	26.3(4.7)	27.3(5.3)
ASA fitness grade			
I	39	21	18
II	93	59	34
III	69	50	19
IV	9	4	5
Missing	12	0	12
History of diverticulitis	54	32	22
History of laparotomy	19	12	7
Corticosteroid use	33	29	4
Insulin use	5	5	0
Smoking	40	19	21

**Table 2:** Baseline characteristics for all patients who had laparoscopic lavage

	Laparoscopic lavage (*n* = 116)	SCANDIV (*n* = 70)	LOLA (*n* = 46)
Age (years), mean(s.d.)	65.0(13.3)	66.8(13.5)	62.4(12.7)
Sex ratio (M : F)	64 : 52	38 : 32	26 : 20
BMI (kg/m^2^), mean(s.d.)	26.8(5.)5	26.5(5.0)	27.5(6.2)
ASA fitness grade			
I	21	11	10
II	57	36	21
III	25	20	5
IV	6	3	3
Missing	7	0	7
History of diverticulitis	28	16	12
History of laparotomy	8	4	4
Corticosteroid use	16	15	1
Insulin use	3	3	0
Smoking	21	8	13

**Table 3:** Baseline characteristics of all patients who underwent resection

	Resection (*n* = 106)	SCANDIV (*n* = 64)	LOLA (*n* = 42)
Age (years), mean(s.d.)	63.7(14.2)	63.6(15.3	64.0(12.3
Sex ratio (M : F)	61 : 45	36 : 28	25 : 17
BMI (kg/m^2^)	26.5(4.4)	26.1(4.)5	27.1(4.4)
ASA fitness grade			
I	18	10	8
II	36	23	13
III	44	30	14
IV	3	1	2
Missing	5	0	5
History of diverticulitis	26	16	10
History of laparotomy	11	8	3
Corticosteroid use	17	14	3
Insulin use	2	2	0
Smoking	19	11	8

In the resection group, patients had either Hartmann's operation (67), or sigmoid resection with (15) or without (18) a stoma. Two patients had an anastomosis that was later converted into Hartmann's operation, and four patients in the resection group underwent lavages. The median age of patients in the Hartmann's group was 69 (i.q.r 58–76) years and that of patients undergoing primary anastomosis was 58 (i.q.r 47–69) years.

The treatment failure rate was 32.8 per cent (38 patients) in the laparoscopic lavage group and 18.9 per cent (20 patients) in the resection group (*P* = 0.019).

In univariable analysis, smoking was a risk factor for treatment failure in the laparoscopic lavage group (OR 3.24, 95 per cent c.i. 1.19 to 8.81; *P* = 0.024), but not in the resection group (OR 0.46, 0.10 to 2.18; *P* = 0.326). The same was true for corticosteroid use in the laparoscopic lavage (OR 4.83, 1.55 to 15.08; *P* = 0.022) and resection (OR 1.88, 0.52 to 6.81; *P* = 0.335) groups. Higher ASA grade was a risk factor for both laparoscopic lavage (OR 3.21, 1.31 to 7.90; *P* = 0.011) and resection (OR 3.30, 1.12 to 9.71; *P* = 0.030) (*Table 4*).

**Table 4:** Univariable logistic regression analysis of risk factors for treatment failure with interaction model for adjustment for study

	Resection		Lavage	
	OR	*P*	OR	*P*
Age (years)	1.04 (0.10, 1.08)	0.086	1.01 (0.98, 1.05)	0.389
Age ≥ 65 years	1.28 (0.48, 3.45)	0.622	2.09 (0.92, 4.75)	0.077
Sex	0.90 (0.33, 2.45)	0.839	1.40 (0.63, 3.08)	0.407
BMI (kg/m^2^)	0.99 (0.88, 1.11)	0.848	0.91 (0.83, 0.10)	0.049
BMI ≥ 25 kg/m^2^	1.04 (0.37, 2.95)	0.935	0.65 (0.28, 1.52)	0.321
ASA fitness grade	3.30 (1.12, 9.71)	0.030	3.21 (1.31, 7.90)	0.011
History of diverticulitis	1.04 (0.33, 3.28)	0.941	0.77 (0.30, 2.01)	0.593
History of laparotomy	3.18 (0.81, 12.49)	0.097	1.13 (0.25, 5.15)	0.871
Corticosteroid use	1.88 (0.52, 6.81)	0.335	4.83 (1.55, 15.08)	0.007
Smoker	0.46 (0.10, 2.18)	0.326	3.24 (1.19, 8.81)	0.022
GI surgeon present during surgical procedure	0.82 (0.24, 2.82)	0.749	1.174 (0.50, 2.73)	0.710
Interval between admission and surgery (h)	1.01 (1.00, 1.02)	0.184	1.00 (0.97, 1.01)	0.197

Values in parentheses are 95% confidence intervals. GI, gastrointestinal.

In multivariable analysis, the only statistically significant risk factors for treatment failure were smoking (OR 7.05, 2.07 to 23.98; *P* = 0.002) and corticosteroid use (OR 6.02, 1.54 to 23.51; *P* = 0.010) in the laparoscopic lavage group (*Table 5*).

**Table 5:** Multivariable logistic regression analysis of risk factors for treatment failure with interaction model for adjustment of study

	Resection		Lavage	
	OR	*P*	OR	*P*
ASA fitness grade	3.11 (0.98, 9.90)	0.055	2.13 (0.69, 6.59)	0.188
BMI (kg/m^2^)	0.99 (0.86, 1.14)	0.895	0.94 (0.85, 1.04)	0.245
Corticosteroid use	0.99 (0.25, 3.97)	0.991	6.02 (1.54, 23.51)	0.010
Smoker	0.42 (0.08, 2.11)	0.288	7.05 (2.07, 23.98)	0.002

Values in parentheses are 95% confidence intervals.

## Discussion

By combining two of the largest randomized trials of treatment of perforated diverticulitis, a large prospectively collected data set within a trial setting of patients with Hinchey III disease could be assessed. Main risk factors for treatment failure in the laparoscopic lavage group were smoking and corticosteroid use. No other factors analysed were associated with treatment failure. Smoking is known to impair wound healing and is a well acknowledged risk factor for complications^18^. Therefore, in elective surgery, smoking cessation is advised possibly in conjunction with increased awareness in postoperative care for these patients as they are at higher risk of complications both in the elective and emergency settings.

Concerns have been raised regarding patients who were diagnosed with cancers and misdiagnosed as having Hinchey III perforated diverticulitis initially. In the SCANDIV study, five patients were assessed as having Hinchey III perforated diverticulitis and three of these were randomized to the laparoscopic lavage arm. Two of these patients had failure of lavage, and both were diagnosed with sigmoid cancer and had surgery within 90 days of laparoscopic lavage (1 sigmoid resection and 1 transversostomy before neoadjuvant treatment for sigmoid cancer with liver metastasis). Of the nine patients who experienced treatment failure after lavage in the LADIES trial, one was diagnosed with an underlying carcinoma during pathological assessment. In both studies, these patients were included in the analysis, as this best reflects clinical practice with the inherent difficulty in differentiating correctly between perforated diverticulitis and perforated carcinoma.

In recent years, trials^19,20^ have compared laparoscopic peritoneal lavage with resectional surgery. Primary anastomosis appears the preferred option over Hartmann's procedure in haemodynamically stable patients as there are fewer stomas and fewer additional procedures, with comparable morbidity and mortality^19,21^. Primary anastomosis, however, is not always easily performed, particularly in the emergency setting or in the absence of a surgeon experienced in emergency colorectal procedures. A recent study^22^ comparing colorectal with non-colorectal surgeons performing primary anastomosis in acute diverticulitis surgery revealed a 1.4 times higher mortality rate if the anastomosis was created by a non-colorectal surgeon. Laparoscopic lavage may potentially serve as a bridge to convert an acute situation to an elective situation, ensuring better treatment outcomes both in the short and long term. Therefore, to minimize the risk of treatment failure in patients undergoing lavage, patient selection has become essential, necessitating risk assessment. In this analysis, the authors chose not to subdivide the resection group into anastomosis with or without stoma and Hartmann's procedure because the groups were heterogeneous. The choice of primary anastomosis or Hartmann's procedure was at the surgeon's discretion in the SCANDIV study, and it is known from clinical practice that younger and more stable patients are usually chosen for primary anastomosis. Such selection bias was demonstrated by the 11-year difference in median age between the groups in this analysis.

Other studies have examined risk factors for failure of laparoscopic lavage. In a study by Radé *et al*.^14^, risk factors for treatment failure following laparoscopic peritoneal lavage were age over 80 years, immunosuppression, and ASA grade above III. In multivariable analysis, only ASA grade over III remained associated with treatment failure. Greilsamer *et al*.^15^ investigated potential risk factors in a cohort of 71 patients with Hinchey III disease, and found immunosuppression to be the only independent predictor of treatment failure from a broad range of factors. Finally, the larger LLO study^16^, which included 231 patients with Hinchey III diverticulitis, reported a short-term failure rate of 25 per cent (within 60 days) after laparoscopic lavage. A Mannheim Peritonitis Index score of at least 24 and ASA grade above III were identified as risk factors for treatment failure that could be assessed before surgery. Furthermore, an increased BMI or higher Mannheim Peritonitis Index score was independently associated with an increased risk of reoperation within the first 60 days after the procedure. The present study adds new information to these potential risk factors and might further aid the decision-making process.

The strength of this study lies in the inclusion of the two largest randomized cohorts studying Hinchey III diverticulitis in a prospective manner. Nevertheless, there are several methodological differences between the SCANDIV and LOLA studies which limit interpretation of the results. For example, the set of baseline characteristics collected and secondary outcomes studied were different. Hence, not all baseline characteristics could be considered in the risk factor analyses. To correct for study heterogeneity in the statistical analyses, care was taken to adjust for study population. As exclusion criteria were different, many patients with high corticosteroid use were excluded from the LOLA study, but were included in the SCANDIV study, making the baseline populations different. Furthermore, both trials did not register Mannheim Peritonitis Index and it was therefore not possible to assess this as a risk factor in the present analysis. Although this is the largest cohort of patients with Hinchey III diverticulitis who underwent laparoscopic lavage in a trial setting, the total number of included patients and variables still limited the extent of multivariable analyses.

In the largest assessment of risk factors for treatment failure of laparoscopic peritoneal lavage in a prospectively collected Hinchey III cohort, active smoking and corticosteroid use at index surgery were found to be statistically significant. Smoking and corticosteroid use are, therefore, factors that should be considered when assessing surgical options for patients with perforated Hinchey III diverticulitis.

## Collaborators

A Papp, U Ersson, T Zittel, N Fagerström, D Gustafsson, G Dafnis,: M Cornelius, M Egenvall, P O Nyström, I Syk, D Vilhjalmsson, C Wallon, G Arbman, J Folkesson, A Chabok, M Helgeland, J Bondi, A Husby, R Helander, A Kjos, H Gregussen, A J Talabani, G Tranø, I H Nygaard, G Wiedswang, O Helmer, K F Desserud, S Norderval, M Vikhammer Gran, T Pettersen, A Sæther, H Kørner; L Blesic, H M Forsmo, S Vennix, GD Musters, IM Mulder, HA Swank, EC Consten, EH Belgers, AA van Geloven, MF Gerhards, MJ Govaert, WM van Grevenstein, A Hoofwijk, PM Kruyt, SW Nienhuijs, MA Boermeester, J Vermeulen, S van Dieren, WC Hop, BC Opmeer, JB Reitsma, RA Scholte, EWH Waltmann, DA Legemate, JF Bartelsman, DW Meijer, M de Brouwer, J van Dalen, M Durbridge, M Geerdink, GJ Ilbrink, S Mehmedovic, P Middelhoek, M J Boom, ECJ Consten, JDW van der Bilt, GDJ van Olden, MAW Stam, MS Verweij, ORC Busch, CJ Buskens, Y El-Massoudi, AB Kluit, C van Rossem, MP Schijven, PJ Tanis, C Unlu, MF Gerhards, TM Karsten, LC de Nes, H Rijna B, A van Wagensveld, GI Koffeman, EP Steller, JB Tuynman, SC Bruin, DL van der Peet, CFJ M Blanken-Peeters, HA Cense, E Jutte, RMPH Crolla, GP van der Schelling, M van Zeeland, EJR de Graaf, RPR Groenendijk, TM Karsten, M Vermaas, O Schouten, MR de Vries, HA Prins, DJ Lips, RJI Bosker, JAB van der Hoeven, J Diks, PW Plaisier, PM Kruyt, C Sietses, MWJ Stommel, SW Nienhuijs, IHJT de Hingh, MDP Luyer, G van Montfort, EH Ponten, JF Smulders, EB van Duyn, JM Klaase, DJ Swank, RT Ottow, HBAC Stockmann, J Vermeulen, RJCLM Vuylsteke, HJ Belgers, S Fransen, EM von Meijenfeldt, MN Sosef, AA W van Geloven, ER Hendriks, B ter Horst, MMN Leeuwenburgh, O van Ruler, JM Vogten, EJ C Vriens, M Westerterp, QAJ Eijsbouts, A Bentohami, TS Bijlsma, N de Korte, D Nio, JJA Joosten, LPS Stassen, MJ Wiezer, EJ Hazebroek, AB Smits, HL van Westreenen, A Brandt, WN Nijboer, BR Toorenvliet, WF Weidema, PPL O Coene, GHH Mannaerts, D den Hartog, RJ de Vos, JF Zengerink, AGM Hoofwijk, KW E Hulsewé, J Melenhorst, JHMB Stoot, WH Steup, PJ Huijstee, JWS Merkus, JJ Wever, JK Maring, J Heisterkamp, WMU van Grevenstein, MR Vriens, MGH Besselink, IHM Borel Rinkes, AJ Witkamp, GD Slooter, JLM Konsten, AF Engel, EGJM Pierik, TG Frakking, D van Geldere, GA Patijn, AJL D’Hoore, A de Buck van Overstraeten, M Miserez, I Terrasson, A Wolthuis, S Di Saverio, MG De Blasiis.

## Data Availability

Original data will be available upon request.
